# Asthmatic condition induced the activity of exosome secretory pathway in rat pulmonary tissues

**DOI:** 10.1186/s12950-021-00275-7

**Published:** 2021-04-01

**Authors:** Asheed Almohammai, Reza Rahbarghazi, Rana Keyhanmanesh, Jafar Rezaie, Mahdi Ahmadi

**Affiliations:** 1 Student Research Committee, Tabriz University of Medical Sciences, Tabriz, Iran; 2 Stem Cell Research Center, Tabriz University of Medical Sciences, Tabriz, Iran; 3 Department of Applied Cell Sciences, Faculty of Advanced Medical Sciences, Tabriz University of Medical Sciences, Tabriz, Iran; 4 Drug Applied Research Center, Tabriz University of Medical Sciences, Tabriz, Iran; 5 Tuberculosis and Lung Diseases Research Center, Tabriz University of Medical Sciences, Tabriz, Iran; 6 Applied Biomedical Research Center, Mashhad University of Medical Sciences, Mashhad, Iran; 7 Solid Tumor Research Center, Cellular and Molecular Medicine Institute, University of Medical Sciences, Urmia, Iran

**Keywords:** Asthma, Exosome biogenesis, CD63, Inflammatory cytokines, Rats

## Abstract

**Background:**

The recent studies highlighted the critical role of exosomes in the regulation of inflammation. Here, we investigated the dynamic biogenesis of the exosomes in the rat model of asthma.

**Results:**

Our finding showed an increase in the expression of IL-4 and the suppression of IL-10 in asthmatic lung tissues compared to the control samples (*p* < 0.05). Along with the promotion of IL-4, the protein level of TNF-α was induced, showing an active inflammatory status in OVA-sensitized rats. According to our data, the promotion of asthmatic responses increased exosome biogenesis indicated by increased CD63 levels and acetylcholine esterase activity compared to the normal condition (*p* < 0.05).

**Conclusion:**

Data suggest that the stimulation of inflammatory response in asthmatic rats could simultaneously increase the paracrine activity of pulmonary cells via the exosome biogenesis. Exosome biogenesis may correlate with the inflammatory response.

## Background

Asthma is a pathologic state of bronchopulmonary tissue with a considerable socioeconomic burden on health care systems and is mainly characterized by chronic inflammation and profound structural remodeling [1]. Based on the released statistics, the prevalence of asthma is high in either developing or industrialized countries [2]. Histopathological changes are integral to the reduction of the Th2/Th1 ratio and accumulation of immune cells in the pulmonary niche [3]. Due to the sustained immune cell infiltration and activation, it has been shown the normal and physiological activity of multiple cell types with distinct functions are altered within the pulmonary tissue [1, 4]. Monitoring early-stage asthmatic changes in the pulmonary niche could help us to predict the asthma progression and prevent further harmful changes in the function of the respiratory tract. In addition to morphological and functional alterations, the detection of paracrine activity and the release of different factors to the airway conduits are also helpful to monitor underlying pathologies [5]. The disproportion inflammatory response in the severe asthma process may comprise various intercellular communication facilitated by signaling molecules such as exosomes [5]. Exosomes, ranging from 30 to 150 nm size nanoparticles, are released from each cell in response to different stimuli under physiological and pathological conditions [6, 7]. These nano-sized particles harbor both genomic and proteomic elements which could reflect the time-lapse changes of the original cells [8, 9]. In addition to the fundamental role of exosomes in cell homeostasis and the removal of exhausted biomolecules, it is noteworthy that the promotion of asthmatic changes could alter the basal dynamic exosome biogenesis in surface respiratory epithelial cells [10]. Along with these changes, different cell types, either recruited immune cells or local bronchopulmonary cells, could release various amounts of exosomes upon initiation of asthmatic changes to regulate in cell-to-cell communication or to signal the occurrence of pathological conditions [10, 11]. Previously, our research group showed enhanced exosome biogenesis and secretion from human mesenchymal stem cells (MSCs) incubated in a culture medium supplemented with diabetic sera, showing the potency of inflammatory conditions to modulate exosome biogenesis [12]. Commensurate with these descriptions, there are some experiments related to the application of stem cell derived exosomes in the alleviation of asthma [9, 10]. It has been suggested that exosomes can transfer specific bioactive factors, which control the intensity and persistence of inflammation by delivering cargo to the target cells [7, 13–15].

Along with the above-mentioned facts, the current study is a preliminary study to analyze the potency of asthma in the modulation of the exosome biogenesis and secretion in the rat model. We hypothesize that the promotion of asthma could alter total exosome biogenesis in an asthmatic niche. Results from the current experiment could help us to predict the occurrence and intensity of immunological response in the asthmatic niche by monitoring exosome biogenesis. In this case, exosomes are suitable as diagnostic tools that could be used as novel asthma-related biomarkers.

## Methods

### Animals and ethical issues

All animals were treated according to the previously delivered guidelines (NIH Publication No. 85-23, revised 1996). Experimental protocols were locally reviewed and approved by the ethics committee of Tabriz University of Medical Sciences (No: IR.TBZMED.VCR.REC.1398.308).

### Experimental protocol

Eight-week-old male Wistar rats (*n* = 16; weighing 200–250 *g*) were subjected to the current experiment. Animals were kept under the standard condition (20–22 °C with a relative humidity of 50 ± 10%). Animals had free access to water and food. Rats were randomly allocated into two groups as follows: control and asthmatic rats (each in 8).

### Sensitization and challenge with OVA

On this basis, we followed the previous protocol that lasts over 32 ± 1 days [16–18]. Briefly, rats received 1 mg OVA (Sigma-Aldrich) via an intraperitoneal route on days 1 and 8. OVA was dissolved in 1 ml normal saline solution containing 200 mg aluminum hydroxide followed by the inhalation of 4% aerosolized OVA via a nebulizer apparatus (Model: CX3, Omron Co., Netherland) from days 14 to 32 ± 1. The inhalation protocol was performed for 5 minutes daily in a whole-body inhalation exposure chamber (Dimensions 30 × 20 × 20 cm^3^). In the control group, normal saline was used as a control vehicle using a similar protocol. After completion of asthma induction, rats from each group were sacrificed humanly and pulmonary tissues were taken soon after rats’ scarification.

### Bronchoalveolar lavage fluid (BALF) preparation

After pectoral incision, BALF was collected immediately by five consecutive 1 mL instillations of sodium chloride 0.9% by a *catheter* connected to each trachea [4].

### Pathological evaluation

To investigate histological changes, lung samples from each group were collected and fixed in a 10% buffer formalin solution (Merck, Germany). Specimens were passaged by serial concentrations of EtOH (70-100%). Then, 4-μm thick sections were prepared and loaded on glass slides. All sections were stained by hematoxylin-eosin (H&E) solution [19]. To confirm the asthmatic condition, we followed the specific pathological changes as follows: leukocyte infiltration, bronchus-associated lymphoid tissue (BALT) hyperplasia, peribronchial cuffing, and emphysema.

### Real-time PCR analysis

The transcription levels of *IL-4 and IL-10* were quantitatively determined by real-time PCR assay according to previously published methods [16, 20]. In brief, 40 mg lung sample was lysed for total RNA extraction using a total RNA extraction mini kit (YTA, Iran). The concentration of total RNA was measured by a NanoDrop 1000 Spectrophotometer (Thermo Scientific, Wilmington, DE 19810 USA). The first-strand cDNA was synthesized from total RNA using a cDNA Synthesis Kit (YTA, Iran). To measure relative mRNA levels, the SYBR Green master mix (YTA, Iran) and Rotor-Gene 6000 instrument (Corbett Life Science, Australia) were used. All samples were measured in triplicate. The relative transcript level of each target gene was normalized to the GAPDH gene using the 2^−∆∆Ct^ method. The list of primer sequences was enlisted in Table 1.

**Table 1:** The list of primers used for real-time PCR analysis

Gene	Primer sequence (***5***'-***3***')	
	Forward	Reverse
IL-4	CTGTCACCCTGTTCTGCTTTCT	CTGGTACAAACATCTCGGTGCA
IL-10	TGAGAATAAAAGCAAGGCAGTGG	GTAGGCTTCTATGCAGTTGATGA
GAPDH	TTG CCA TCA ACG ACC CCT TCA	AGC ACC AGC ATC ACC CCA TTT

### Enzyme-linked immunosorbent assay (ELISA)

We used an ELISA to determine the protein level of TNF-α in bronchoalveolar lavage fluid (BALF) using Rat TNF-α ELISA kit (Cat. no: EK0526). Briefly, we incubated each well of 96-well plates with 100 μl BAL samples from each group and kept at 37 °C for 90 min. Then, the solution was discarded and 100 μl working solution was added and incubated at 37 °C for 60 min. The solution was discarded and washed twice in phosphate-buffered saline (PBS). After PBS wash, 100 μl ABC working solution was added to each well and incubated at 37 °C for 30 min. Then, wells were washed 5 times in PBS, and 90 μl chromogen substrate 1,2,4,5- tetramethyl benzene (TMB) was poured into each well and kept for 20 min. To stop the reaction, 100 μl stop solution was added to each well. The absorbance was recorded after 30 min using a microplate reader (Biotek) at 450 nm.

### Western blotting

The protein levels of CD63, a protein involved in exosomes biogenesis [21], were evaluated by western blotting analysis. For this purpose, 50 mg of lung tissue were lysed in RIPA lysis buffer supplemented with a protease inhibitor cocktail (Sigma Aldrich) and then homogenized with an electric homogenizer. The lysates were obtained by centrifugation at 12,000*g* for 20 min at 4 °C. In brief, 100 μg of each sample lysates were separated by 10% sodium dodecyl sulfate-polyacrylamide gel electrophoresis (SDS-PAGE) and transferred to 0.2-μm polyvinylidene difluoride (PVDF, 249 Millipore) membranes. After blocking with 5% skim milk (Gibco), membranes were incubated with primary anti-human CD63 antibody (Cat no: sc-5275) overnight at 4 °C followed by incubated with secondary antibody (goat anti-mouse IgG-HRP: DB9571, Iran) 1 h at 37 °C. Immunoblotting bands were visualized using a chemiluminescence (ECL) detection system according to the manufacturer’s instructions (Roche). β-actin (Cat no: sc-47778) was kept as a housekeeping protein for normalization. Relative protein levels were calculated using NIH ImageJ software ver. 1.44p.

### Exosome isolation

After treatment, BAL samples were collected and after removing debris at 300 g for 10 min, exosomes were isolated using Exo-spin™ kit (EX01-8, Cell Guidance Systems) according to the manufacturer’s guidelines. The proper volume (1/2 ratio) of Exo-spin™ buffer was mixed with each sample and kept overnight at 4 °C. Next, the mixtures were centrifuged at 16,000×*g* for 1 h using the provided filter columns. Exosome pellets were suspended in 100 μl PBS and kept at 4 °C.

### Characterization of exosomes

Exosomes were subjected to transmission electron microscopy (TEM) and flow cytometry analysis. For TEM analysis, 20 μl exosome suspension were fixed with glutaraldehyde 1%, and then loaded on the carbon grids at room temperature and were allowed to dry. After twice washing, exosomes were stained with uranyl acetate 1% for 10 min at room temperature and were visualized using the TEM system (Philips BioTwin, CM100, The Netherlands) at 80 kV. For flow cytometry analysis, 200 μl exosome suspension was incubated with the primary anti-CD63 antibody (Cat no: sc-5275) for 2 h at 4 °C. Next, secondary anti-mouse IG-FITC (cat no: 406001, BioLegend) was added and incubated at 37 °C for 1 h. Samples were subjected to a BD FACSCalibur system and analyzed by FlowJo software (version 7.6.1).

### Quantification of exosomes

To quantity the number of exosomes in BAL samples, we performed the acetylcholine esterase assay (AChE activity) using a commercial cholinesterase kit (Cat no. BXC080, Iran) according to the company’s guidelines. In brief, 100 μl of BAL samples were mixed with 500 μl R1 buffer (pyrophosphate + potassium hexacyanoferrate) and kept for 5 min at room temperature. Then, 20 μl R2 buffer (S-butyryl thiocholine iodide) was added, and optical density was obtained at 405 nm by three different intervals using a microplate reader system (BioTek). AhCE activity was measured by the following formula: Activity (U/l) = 65,800 × ΔAbs/min.

### Data analysis

All quantitative results were analyzed using a student *t*-test and presented as mean ± SEM. Statistical significance was set at *p* values of < *0.05*.

## Results

### Pathological changes were indicated in the asthmatic pulmonary niche

**Fig. 1:**
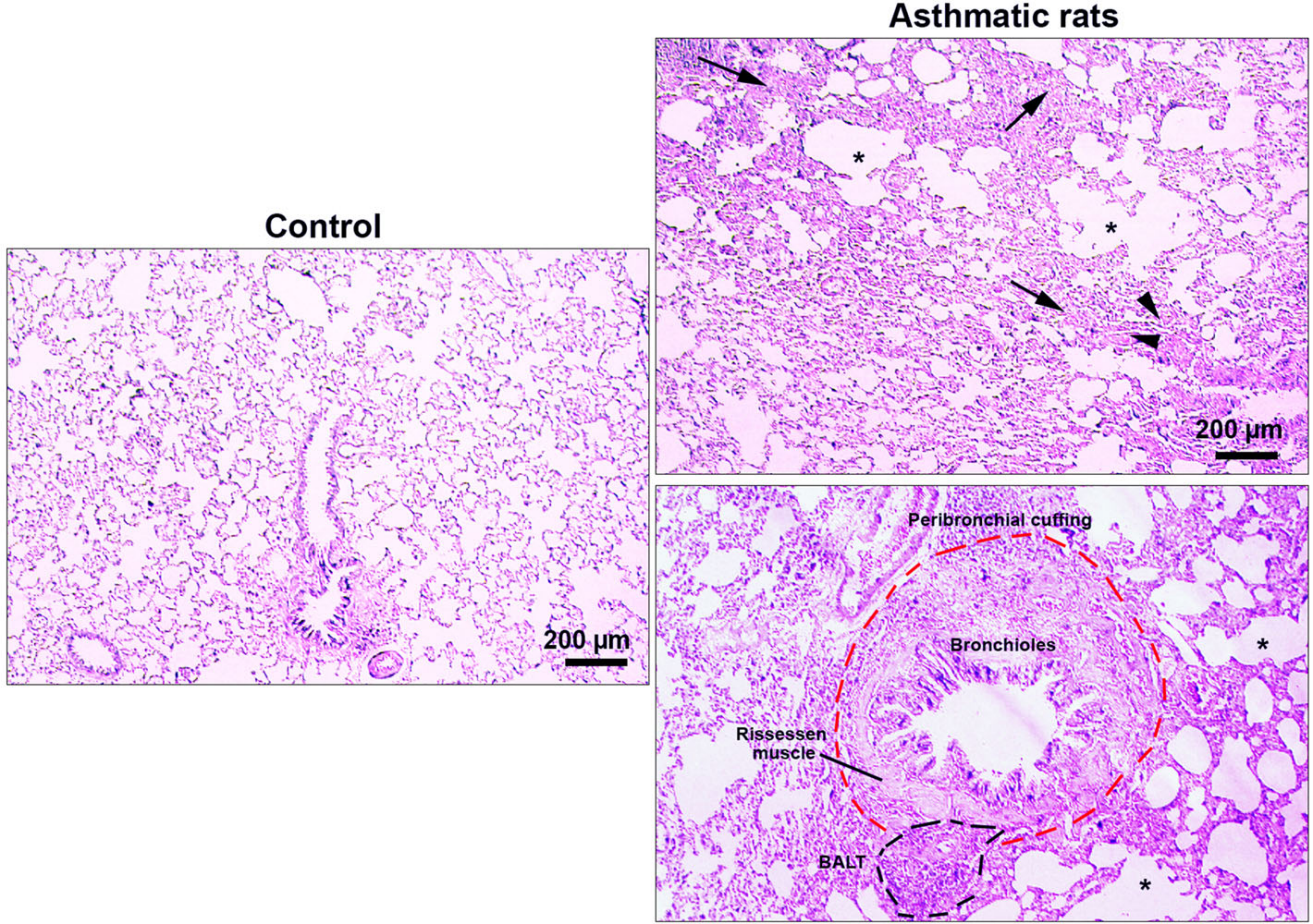
Histopathological examination of asthmatic pulmonary tissue using H&E staining. Asthmatic changes were indicated by prominent interstitial pneumonia (arrows), focal hemorrhagia (arrows heads), and emphysema. The pathological remodeling coincided with the peribronchial cuffing, bronchial smooth muscle hypertrophy, and BALT hyperplasia. These features highlight the efficiency of our protocol in the induction of asthmatic changes using OVA.

To confirm asthmatic changes, we performed histopathological analysis on pulmonary tissues obtained from the control and asthmatic lungs. Data revealed, massive leukocyte infiltration, bronchus-associated lymphoid tissue (BALT) hyperplasia, peribronchial cuffing, and emphysema in lung tissues of asthmatic rats compared to the control rats, indicating the efficiency of our protocol to induce asthmatic change established (Fig. 1).

### IL-4 and IL-10 expression was changed in the asthmatic lung tissue

**Fig. 2:**
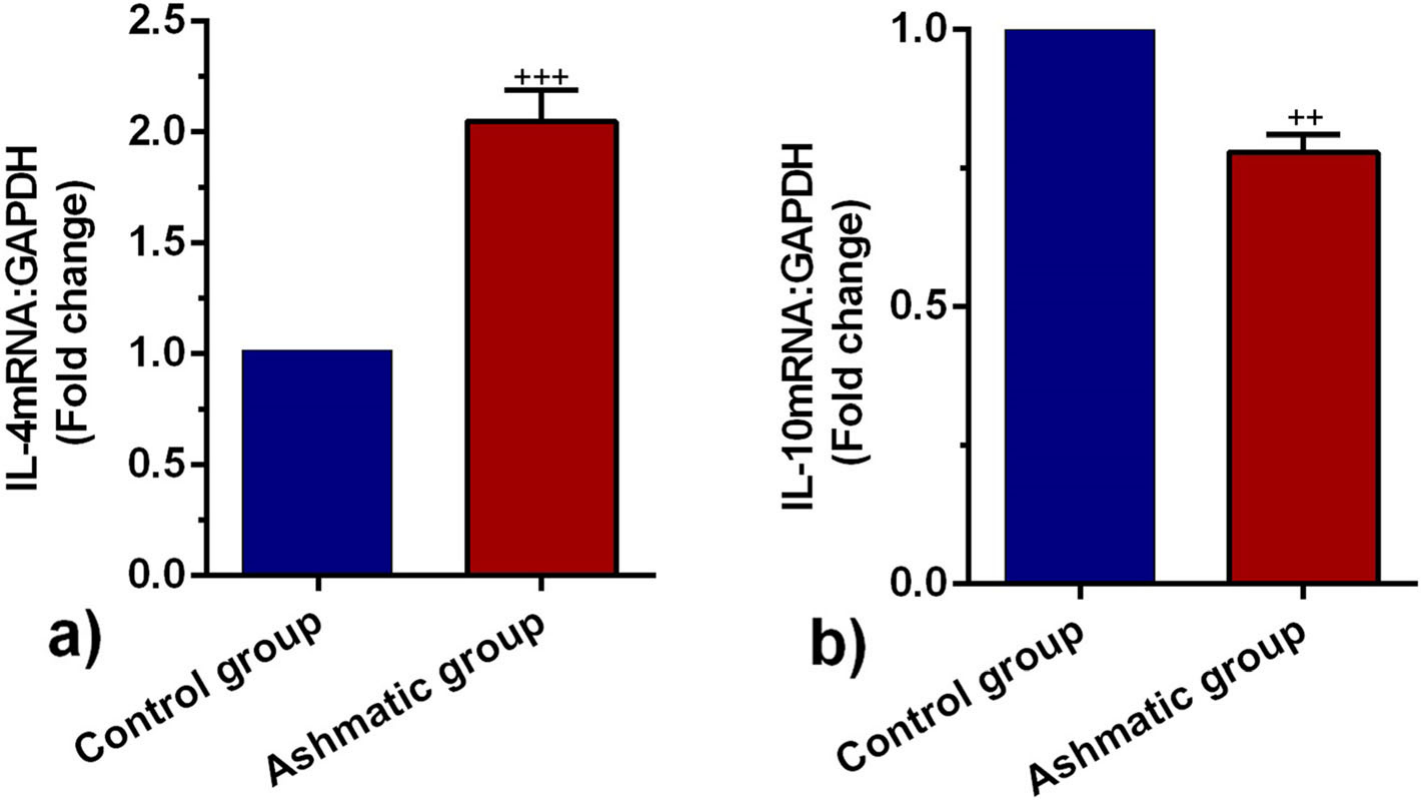
Measuring the transcription of *IL-4* **a** and *IL-*10 **b** mRNA in the lung tissues of control and asthmatic group (*n* = 8). Bars represent the mean ± SEM. Statistical differences between control and asthmatic group: ++; *p* < 0.01 and +++; *p* < 0.001

To observe the inflammatory status of asthmatic lungs, we monitored the expression of two interleukins IL-4 and IL-10 by real-time PCR assay. According to our data, the expression of IL-4 and IL-10 were changed significantly in the asthmatic group as compared to healthy rats (*p* < 0.05; Fig. 2a, b). In this regard, we showed enhanced expression of IL-4 coincided with suppression of IL-10 in asthmatic rats as compared with the control group (from *p* < 0.001 to *p* < 0.01 respectively) (Fig. 2a, b). The increase of IL-4 and suppression of IL-10 is a valuable tool to show the promotion of asthma in rats sensitized to OVA.

### Level of TNF-α was increased in the asthmatic BALF

**Fig. 3.**
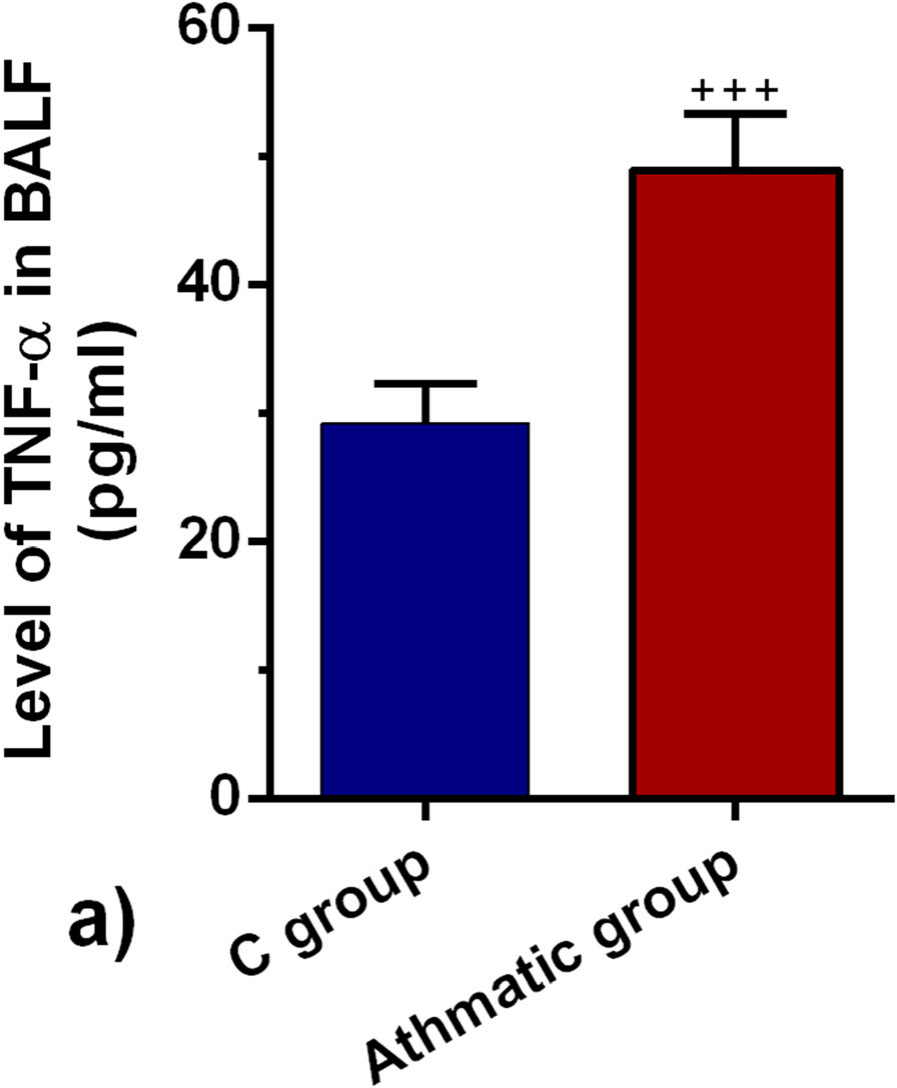
TNF-α in BALF of control and asthmatic rats (*n* = 8). Bars represent the mean ± SEM. Statistical differences between control and asthmatic groups: +++; *p* < 0.001

We also monitored the protein levels of TNF-α in the asthmatic discharge along with the gene expression of IL-10 and IL-4. ELISA showed that the level of TNF-α in BALF was prominently increased in the asthmatic rats in comparison with the control rats (*p* < 0.001; Fig. 3). This data showed that the levels of TNF-α increases with asthma progression.

### CD63 translation was induced in the lung tissues of asthmatic rats

**Fig. 4:**
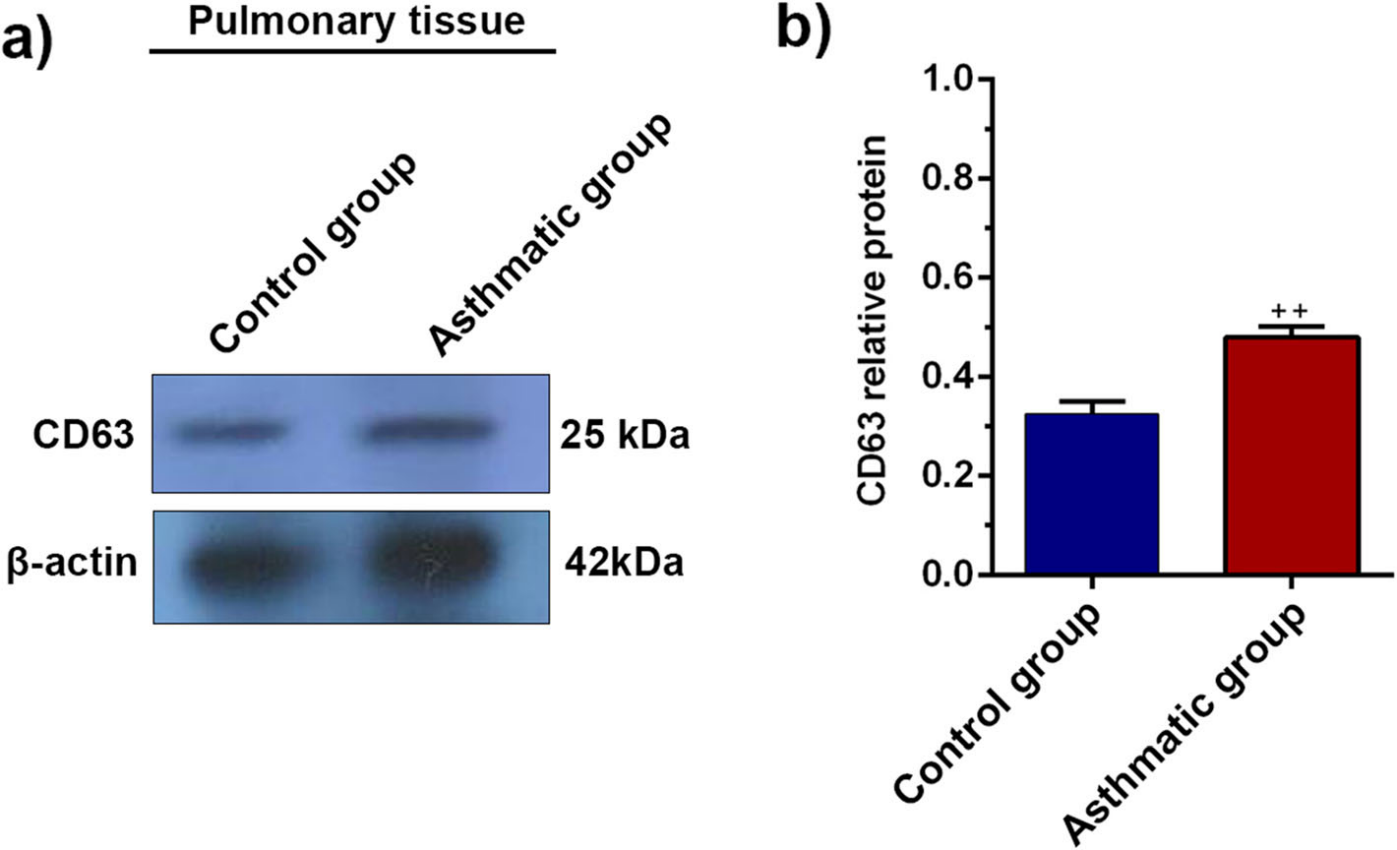
Western blotting analysis of CD63 protein in lung tissues **a**. The relative expression of CD63 protein increased in asthmatic lung tissues **b**. Bars represent the mean ± SEM. Statistical differences between control and asthmatic group: ++; *p* < 0.01

We also analyzed the expression of CD63 protein in the lung tissues of asthmatic rats using western blotting (Fig. 4a, b). Data showed that the protein level of CD63 was increased in asthmatic rats (~ > 0.5–fold) as compared to control rats (*P* < 0.01). These data showed enhanced factor synthesis, which correlated with exosome biogenesis and production

### Enhanced AChE activity was obtained in asthmatic BALF

**Fig. 5:**
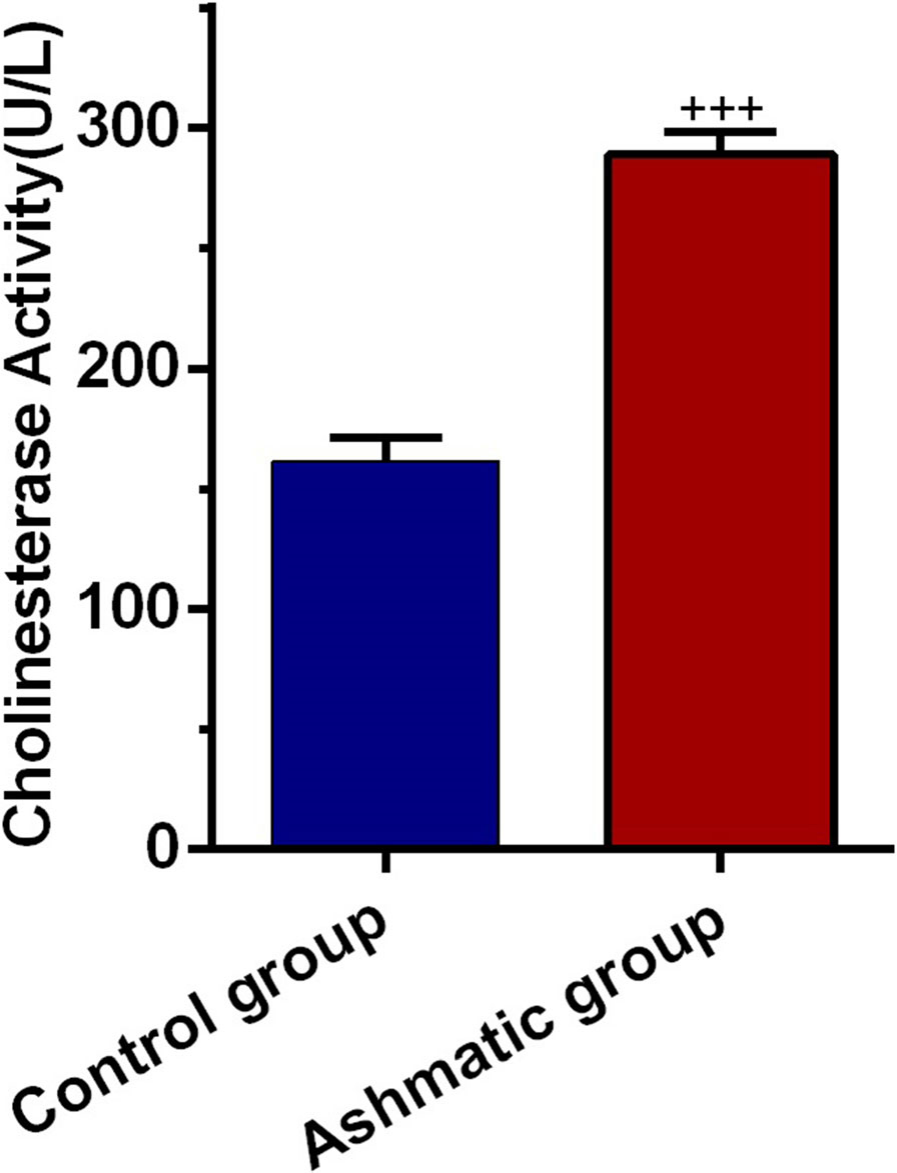
Quantification of the number of exosomes secreted into BAL fluid by acetylcholinesterase (AChE) activity assay. Bars represent the mean ± SEM. Statistical differences between control and asthmatic group: +++; *p* < 0.001

By monitoring the activity of AChE, we also indirectly showed exosome release to the BALF. Based on data, a significant increase was found in the AChE activity of asthmatic BALF as compared to control samples (*P <* 0.001, Fig. 5). The data highlighted a prominent AChE activity in asthmatic discharge, which could be related to the paracrine activity of cells exposed to asthmatic changes.

### Characterization of purified exosomes

**Fig. 6:**
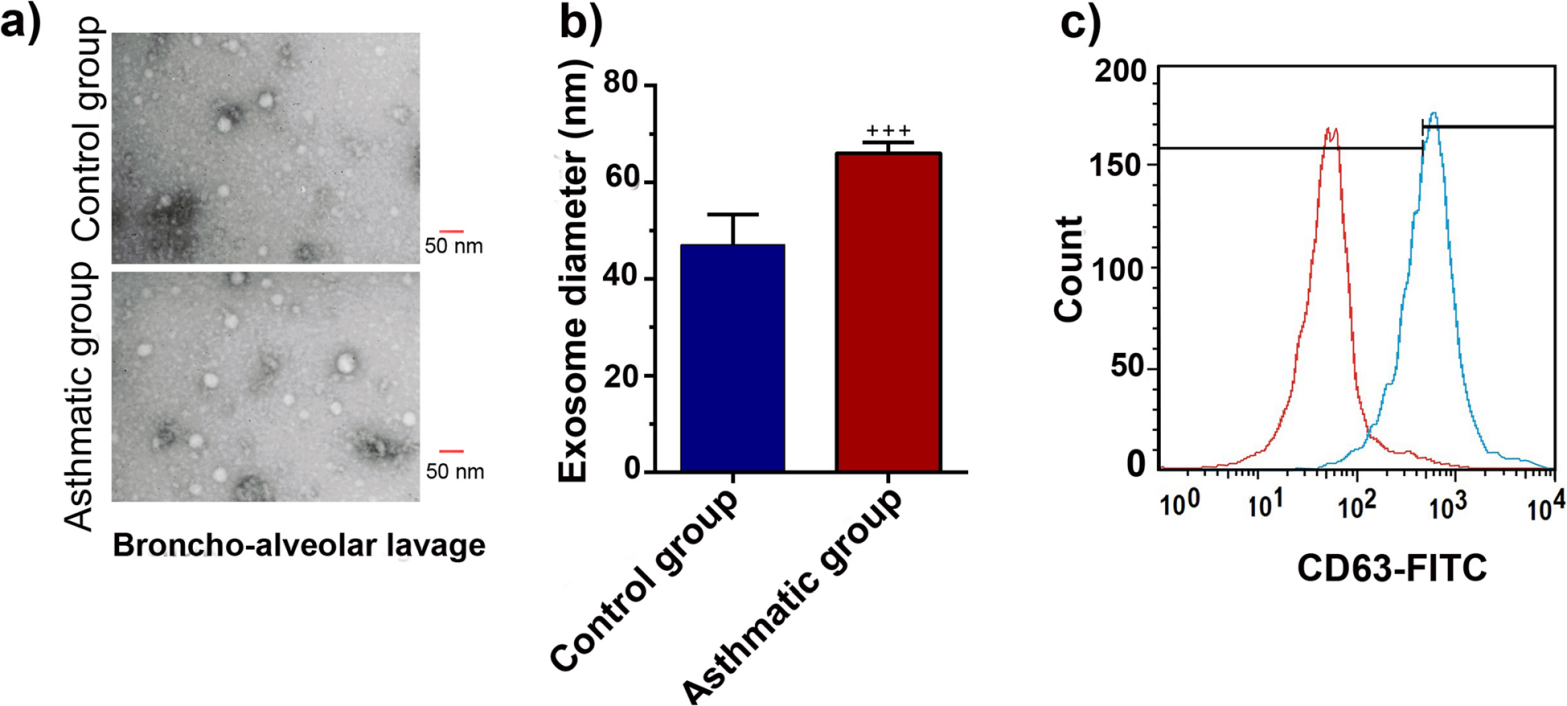
Transmission electron micrographs of isolated exosomes **a**. Analyzing the size of exosomes diameter purified from BAL samples of asthmatic and control groups **b**. Flow cytometry confirmed the expression of CD63 marker on exosomes **c**. Bars represent the mean ± SEM. Statistical differences between control and asthmatic group: +++; *p* < 0.001

We characterized exosomes released from lung tissue into BALF according to the International Society for Extracellular Vesicles (ISEV) guidelines by TEM and flow cytometry analysis. TEM micrographs indicated a round shape and nano-scale-sized exosomes (Fig. 6a, b). Flow cytometry immunophenotyping confirmed the CD63 marker in purified exosomes (Fig. 6c). Data analysis showed that the diameter of exosomes isolated from asthmatic BAL was significantly increased compared to the control counterparts (*p* < 0.001; Fig. 6b). It seems that the promotion of an active inflammatory response could alter the production and physiochemical properties of exosomes.

## Discussion

This study was conducted to address the impact of the asthmatic condition on the dynamic biogenesis of exosomes in the rat model. Data showed OVA sensitization promoted asthma-like condition in the rats after 32 days. In addition to pathological changes, the level of inflammatory cytokines was also altered. We found that asthmatic changes increased leukocyte infiltration, which contributes to interstitial bronchopneumonia. Along with these changes, hyperplastic BALT, prominent peribronchial cuffing, and emphysema were also evident in the asthmatic niche [16, 18, 22]. It has been thought that the sustained immune cell recruitment correlated with the increase of IL-4 and suppression of Treg lymphocytes [23, 24]. The secretion of IL-4 triggers central pro-inflammatory responses that regulate eosinophil trans-endothelial migration and IgE production and increases the level of endothelial adhesion molecules such as vascular cell adhesion molecule-1 (VCAM-1). Also, IL-4 can increase mucus secretion, and also accelerate the differentiation of T helper type 2 lymphocytes which leads to cytokine secretion [25, 26]. Conversely, we found that the mRNA level of IL-10 was decreased in lung tissues of asthmatic rats, showing an inhibited Treg activity [27]. Our results share several similarities with previous findings that in asthma conditions, secretion of IL-10 was suppressed, which, in turn, induces the production of many pro-inflammatory cytokines [28, 29]. Besides, ELISA has further confirmed the pro-inflammatory condition in lung tissues of asthmatic rats in that, the production of TNF-a protein in BALF samples was increased. In agreement with previous experiment, it seems that the initiation of asthmatic condition triggers a cascade of cytokine release [30, 31]. TNF-a, a proinflammatory cytokine, plays key roles in the modulation of inflammation in different diseases, like asthma [32], and also in exosome biogenesis [33].

To investigate the dynamic of the exosomal secretory pathway in lung tissues of asthmatic rats, we measured the protein level of CD63. Results from western blotting confirmed that the protein level of CD63 was upregulated in asthmatic lungs. CD63, a tetraspanin protein located on the multivesicular body (MVB) and exosome membranes, is involved in the regulation of exosome biogenesis and loading [34]. Paredes et al. found that exosomes collected from patients BALF with mild allergic asthma abundantly contain CD63 protein compared to those from healthy individuals [35]. Besides, we found that the activity of AChE, an exosome-linked enzyme, was increased in BAL samples of asthmatic rats, indicating an increase in exosome secretion. Our observation showed that asthmatic BALs had a higher level of exosomes in BAL fluid than healthy samples aligns with previous reports [35]. The increased secretion of exosomes may either be due to increased formation of exosomes by airway epithelial cells of asthmatics or amplified immune cells infiltrated in the airways of asthmatics [36]. A growing body of evidence indicates cells under such stress conditions have a higher exosome production capacity. Furthermore, immune cell infiltration is well documented in the airways of asthmatics [37]; those release exosomes, participating in inflammatory responses [38]. Besides, it seems likely that the upregulated expression of IL-4 and TNF-a correlates with an increased level of exosome secretion in asthmatic rats [39, 40]. These results show a close association of the exosome biogenesis and secretion with the synthesis of pro-inflammatory cytokines, especially TNF-a when asthma is induced [5, 13]. The increased exosome biogenesis could correlate with the fact that injured cells tend to find a way to eliminate damaged compounds and unwanted proteins by using exosomes and exocytosis [41, 42]. A study by Takahashi et al., for example, revealed that exosome secretion contributes to supporting cell homeostasis by eliminating damaged DNA molecules from inside cells [43]. Whether asthma can alter the paracrine activity of pulmonary tissues by regulation of exosome secretion needs more investigations. We found that exosome size was increased in asthmatic BALFs, indicating the change and alteration in the exosome biogenesis and sorting machinery. To our knowledge, this finding is preliminary result and there are little details about the effect of asthmatic condition on size of exosomes. Similarly, it was demonstrated that the size of exosomes derived from diabetic MSCs was larger than those of secreted from normal MSCs [12]. Bagheri and co-worker previously showed that the size of exosomes did not alter in injured endothelial cells after exposure to laser irradiation [44]. Previous studies showed that cells may form various subpopulations of MVB/exosomes or respond differently to the insulting conditions [45–47]. Therefore, *it can thus be* conceivably hypothesized that asthmatic conditions may change MVB loading/biogenesis pathways that, in turn, affect exosomes size [48]. Therefore, additional studies are essential to elucidate whether increased exosome secretion rate in severe asthmatics condition is a way to decrease the intensity of pathological remodeling or it supports the elimination of exhausted intracellular molecules. Collectively, we showed that upon an increase in pro-inflammatory cytokines and damage in asthmatic lung tissues, exosome secretion, biogenesis are stimulated.

## Conclusion

Taken together, our data indicate that the exosome secretory pathway concurrently with proinflammatory cytokines production was over-activated in the asthmatic lung tissues. Even so, further scrutiny is essential to uncover the key role of asthmatic-induced over-activation in the dynamic of exosome activities and a better understanding of inflammatory mediated by exosomes to improve the outcome of therapies.

## Data Availability

The datasets used and/or analyzed during the current study are available from the corresponding author on reasonable request.

## Abbreviations

AChE: Acetylcholine esterase
BALF: Bronchoalveolar lavage fluid
ECL: Chemiluminescence
ELISA: Enzyme-linked immunosorbent assay
ISEV: International Society for Extracellular Vesicles
MSCs: Human mesenchymal stem cells
MVB: Multivesicular body
PVDF: Polyvinylidene difluoride
TMB: Tetramethyl benzene
VCAM-1: Vascular cell adhesion molecule-1
